# Mitozolomide-induced sensitisation of mammalian cells in vitro to radiation.

**DOI:** 10.1038/bjc.1989.247

**Published:** 1989-08

**Authors:** M. J. McKeage, P. B. Roberts

**Affiliations:** Oncology Department, Wellington Hospital, New Zealand.


Br. J. Cancer (1989), 60, 182–184 © The Macmillan Press Ltd., 1989

SHORT COMMUNICATION

### Mitozolomide-induced sensitisation of mammalian cells *in vitro* to radiation

M.J. McKeage[1] & P.B. Roberts[2]

[1]*Oncology Department, Wellington Hospital, Wellington, New Zealand; and* [2]*Institute of Nuclear Sciences, DSIR, PO Box 31-312 Lower Hutt, New Zealand.*

The synthesis of mitozolomide was reported by Stevens *et al.* (1984) and pre-clinical trials have indicated that it possesses cytotoxic activity against a number of animal tumours (Hickman *et al.*, 1985). Phase II clinical studies have shown moderate antitumour activity (Gunderson *et al.*, 1987), and an early phase clinical study with autologous bone marrow rescue is in progress at Wellington Hospital. In the Wellington Hospital study one patient underwent a course of radiotherapy following treatment with mitozolomide. The patient had a urethral carcinoma which did not respond to mitozolomide, but after radiotherapy the lesion regressed dramatically. This chance observation led us to consider the possibility that mitozolomide had acted as a radiation sensitiser.

It has been suggested that mitozolomide cytotoxicity is due to DNA interstrand cross-linkage, following alkylation of guanine bases (Gibson *et al.*, 1984*a*,*b*). In this respect mitozolomide appears similar to BCNU (1,3 *bis* (2-chloroethyl)-1-nitrosourea) and other nitrosoureas (Kohn, 1977). Radiation can enhance interstrand cross-linking by BCNU (Tofilon *et al.*, 1984). Cross-linking agents have also been found to enhance radiation-induced cell killing in mammalian cells *in vitro* (Nias, 1985; Wheeler *et al.*, 1977) and the combination can be more effective *in vivo* than the single agents (Barker *et al.*, 1979; Walker & Gehan, 1976).

We have therefore investigated the hypothesis that mitozolomide enhances radiation damage using mammalian cells *in vitro*. Chinese hamster ovary (AA8 sub-line) cells were maintained at 37°C as monolayer cultures in minimal essential medium (alpha-modification) with 10% fetal calf serum and without added antibiotics. Sub-culture was carried out bi-weekly. Once cells in late log phase had been trypsinised for use in experiments the fetal calf serum was reduced to 5% and penicillin ($100\,\mu g\,ml^{-1}$) and streptomycin ($100\,\mu g\,ml^{-1}$) added. This latter medium was also used to plate out and incubate the cells.

Mitozolomide was provided by Rhone Poulenc (NZ) Ltd and made up just prior to use by dissolving the powder in dimethylsulphoxide and subsequent dilution into growth medium. The maximum dimethylsulphoxide concentration in the cultures (0.7% v/v) had no effect on cell viability. Cells were exposed to the drug for 1 h, usually by careful addition of the drug to the monolayer cultures. However, in a few experiments the drug was added to cells in suspension. Drug toxicity was similar in monolayer and suspension culture.

After drug exposure the medium was removed and the cells rinsed with medium and either trypsinised immediately or re-incubated with fresh, drug-free medium for a given period before trypsinisation. The cells were centrifuged and resuspended at about $10^6$ cells $ml^{-1}$ in the complete medium. After 20 min re-equilibration at 37°C in an atmosphere of 5% $CO_2$ 95% air, the cells were irradiated in small stoppered glass vessels. The irradiation source (AECL, Gammacell 220) provided a gamma-ray dose rate of about $7\,Gy\,min^{-1}$.

Correspondence: P.B. Roberts.
Received 3 January 1989, and in revised form, 21 March 1989.

Standard methods were used to dilute, plate, incubate and count the surviving cells. Each data point was obtained from triplicate plates and experiments were repeated independently 2–4 times. Untreated cells and cells exposed to drug alone were used as controls in each experiment. The plating efficiency (the number of colonies observed/number of cells plated) was calculated for untreated cells ($PE_c$), and for cells treated by radiation alone ($PE_R$), drug alone ($PE_D$) or drug plus radiation combinations ($PE_{D+R}$).

The fraction of cells surviving treatment with drug, radiation or the combination could be obtained by $PE_D/PE_c$, $PE_R/PE_c$ or $PE_{D+R}/PE_c$ respectively. Experiments were designed principally to test the effect of drug exposure on the radiation response. Therefore the surviving fraction in combination experiments was corrected for the cell killing introduced by the drug alone. Calculation of $PE_{D+R}/PE_D$ yielded the fraction of cells surviving the irradiation in combination experiments for comparison with the results of true radiation-only experiments.

Radiation survival curves were obtained from the data by applying the linear-quadratic equation

$$-\log_e S = \alpha D + \beta D^2, \qquad [1]$$

where $S$ is the fractional survival after a dose $D$, and $\alpha$ and $\beta$ are the fitted coefficients.

Untreated AA8 cells routinely had a plating efficiency, $PE_c$, in the range 0.75–0.90. The toxicity of a 1 h treatment with mitozolomide in the concentration range 30–$50\,\mu mol\,dm^{-3}$ reduced the fraction of viable cells to 0.4–0.15. Figure 1 indicates that when irradiated 8 h later the cells surviving the drug treatment were more sensitive to radiation than non-treated controls ($P<0.005$).

Lower concentrations had a greatly reduced toxic effect. Mitozolomide at $10\,\mu mol\,dm^{-3}$ resulted in a surviving fraction of about 0.8 and 8 h later did not produce any radiosensitisation of the cells which survived the drug treatment ($P>0.3$).

The enhancing effect of the drug was measured by the ratio of doses required to achieve a given surviving fraction in control and drug-treated cells. This enhancement ratio (ER) is $1.25\pm0.04$ throughout the survival range 0.2–0.001 for the curves in Figure 1 fitted by equation [1]. Thus mitozolomide pre-treatment appears to act as a simple dose modifying agent.

The importance of the scheduling of the drug and radiation treatments was also investigated (Figure 2). Enhancement was observed when the radiation exposure followed drug treatment by 4–12 h. Longer or shorter delays before irradiation resulted in almost no enhancement. The comparatively greater error bars at 4 h in Figure 2 reflect the greater variation in the degree of enhancement found at this time. Separate experiments showed that allowing mitozolomide to remain in contact with the cells during the irradiation had no effect on the radiation response.

A number of explanations for our results are possible. In view of the need for toxic drug concentrations and the

**Figure 1** The radiation response of control AA8 cells (□) and cells treated for 1 h with 30 $\mu$mol dm$^{-3}$ (●) or 50 $\mu$mol dm$^{-3}$ (○) mitozolomide. Irradiation was performed in air at room temperature 8 h after completion of drug treatment. The standard error of the mean individual points was almost always within the size of the points. There was no significant difference between the data obtained with the two drug concentrations. The data for drug-treated cells (five separate experiments in total) were therefore pooled and fitted to equation [1]. Values (with standard deviations) for drug-treated cells were $\alpha=0.316$ (0.0297) Gy$^{-1}$ and $\beta=0.0300$ (0.00308) Gy$^{-2}$. Values for control cells were $\alpha=0.208$ (0.0242) Gy$^{-1}$, $\beta=0.0246$ (0.00234) Gy$^{-2}$. The error bars on the fitted curves represent the 95% confidence intervals for the fit.

**Figure 2** The dependence of the enhancement ratio (ER) upon the interval between drug and radiation treatments. Data are represented by the mean and range of values in two or more separate experiments.

passage of several hours before enhancement was observed, the simplest explanation is that the drug treatment altered the distribution and progression of cells within the cell cycle. Between 4 and 12 h after treatment the cells destined to survive the drug exposure are presumed to be distributed throughout the cell cycle stages so as to be more sensitive to radiation than the original asynchronous population. Differences in sensitivity to radiation at different cell cycle stages are well established (Terasima & Tolmach, 1963; Sinclair & Morton, 1966). The results indicate that the alterations in cell cycle distribution important to overall radiation sensitivity occur with drug concentrations between 10 and 30 $\mu$mol dm$^{-3}$, with little further change to 50 $\mu$mol dm$^{-3}$.

Horgan *et al.* (1983) have reported briefly on a flow cytometric analysis of mitozolomide-treated cells. Mitozolomide under conditions described as minimally toxic depleted the $G_1$ cell fraction and produced a block in the cell cycle at $G_2$/M. Broggini *et al.* (1986) reported similar results with mitozolomide treatment of tumour-bearing mice (10 mg kg$^{-1}$). Cell cycle effects less than 24 h after treatment were not examined, but appeared to peak about 48 h after treatment. Although the experimental conditions differed from ours, the flow cytometry results lend some support to an explanation of our results based on cell cycle effects. However, the results of Broggini *et al.* (1986) indicate that the time course of radiosensitisation may differ from cell cycle effects.

Gibson *et al.* (1984*a*,*b*) noted that maximum DNA cross-linkage occurred some 12 h after mitozolomide treatment of a transformed cell line. This is near the peak of the period in which radiation enhancement occurred. It is not possible at present to tell whether this is a coincidental finding or an indication that DNA cross-linking and radiation enhancement are directly related. However, a normal cell line, which was proficient in repair of the initial guanine adducts, demonstrated little cross-linking.

Another possible explanation is that mitozolomide interferes with the accumulation or repair of sub-lethal or potentially lethal damage. This was the explanation tentatively favoured for the enhancement brought about by BCNU in rat 9L brain tumour cells *in vitro* (Wheeler *et al.*, 1977). Since BCNU is thought to have similarities with mitozolomide in its cytotoxic mode of action (Gibson *et al.*, 1984*a*; Horgan & Tisdale, 1984), a similar explanation for mitozolomide should be considered. The maximum enhancement by BCNU at surviving fractions of 0.1 or less is similar to the enhancement by mitozolomide, and this maximum effect also occurred some 5–15 h after BCNU treatment.

However, the differences between the two drugs are even more notable. BCNU provided enhancement at relatively non-toxic concentrations and with a far wider range of schedules than found with mitozolomide, including simultaneous treatments and irradiation before drug treatment. The principal effect of BCNU, seen with all schedules, was to reduce the shoulder on the radiation survival curve, whereas enhancement by mitozolomide was reasonably constant throughout the dose range.

Phase 1 trials of mitozolomide have indicated peak plasma levels in the approximate range 1–10 mg l$^{-1}$ at tolerable doses (M.J. McKeage, unpublished observations; Newlands *et al.*, 1985). Peak tumour levels of 3–15 mg kg$^{-1}$ have been reported in an animal tumour (Broggini *et al.*, 1986). These values may be compared with media concentrations of 30–50 $\mu$mol dm$^{-3}$ or 7–12 mg l$^{-1}$ at which radiosensitisation was observed.

We conclude that mitozolomide has the potential to enhance radiation damage, and this should be borne in mind for radiotherapy patients likely to undergo a concurrent course of mitozolomide. However, our present results suggest that any effect will be modest and be maximal at about 12 h after drug treatment. Effects could be comparable to any produced by BCNU. In the clinically relevant radiation dose range, however, it is more likely that mitozolomide would be less effective than BCNU since the latter is particularly effective in the low dose region and at non-toxic concentrations. The excellent response of a patient to radiotherapy following a course of mitozolomide occurred with a gap of several days between drug and radiation treatments and was probably a chance finding.

Useful discussions with Dr P.J. Dady were much appreciated. Excellent technical assistance was provided by D.M. Chambers. Mitozolomide was a gift from Rhone Poulenc (NZ) Ltd.

### References

BARKER, M., DEEN, D.F. & BAKER, D.G. (1979). BCNU and X-ray therapy of intracerebral 9L rat tumours. *Int. J. Radiat. Oncol. Biol. Phys.*, **5**, 1581.

BROGGINI, M., ERBA, E., MORASCA, L. and 2 others (1986). *In vivo* studies of the novel anticancer agent mitozolomide (NSC 353451) on Lewis lung carcinoma. *Cancer Chemother. Pharmacol.*, **16**, 125.

GIBSON, N.W., ERICKSON, L.C. & HICKMAN, J.A. (1984*a*). Effects of the antitumour agent 8-carbamoyl-3-(2-chloroethyl) imidazo [5, 1-*d*]-1,2,3,5-tetrazin-4(3H)-one on the DNA of mouse L1210 cells. *Cancer Res.*, **44**, 1767.

GIBSON, H.W., HICKMAN, J.A. & ERICKSON, L.C. (1984*b*). DNA cross-linking and cytotoxicity in normal and transformed human cells treated *in vitro* with 8-carbamoyl-3-(2-chloroethyl) imidazole [5,1-*d*]-1,2,3,5-tetrazin-4($^3$H)-one. *Cancer Res.*, **44**, 1772.

GUNDERSON, S., AAMDAL, S. & FOSTAD, O. (1987). Mitozolomide, a new active drug in the treatment of malignant melanoma. Phase II trial in patients with advanced disease. *Br. J. Cancer*, **55**, 433.

HICKMAN, J.A., STEVENS, M.A., GIBSON, N.W. and 6 others (1985). Experimental antitumour activity against murine tumour model systems of 8-carbamoyl-(2-chloroethyl)imidazo[5, 1-*d*]-1,2,3,5-tetrazin-4($^3$H)-one (mitozolomide), a novel broad-spectrum agent. *Cancer Res.*, **45**, 3008.

HORGAN, C.M.T. & TISDALE, M.J. (1984). An investigation into the mechanism of the antitumour activity of a novel and potent antitumour agent, mitozolomide. *Biochem. Pharmacol.*, **33**, 2185.

HORGAN, C.M.T., TISDALE, M.J., ERBAN, E., D'INCALCI, M. & PEPE, S. (1983). Flow cytometric analysis of DNA distribution in lewis lung carcinoma cells after treatment with CCRG 81010 (M and B 39565). *Br. J. Cancer*, **48**, 139.

KOHN, K.W. (1977). Interstrand crosslinking by BCNU and other 1-(2-haloethyl)-1-nitrosoureas. *Cancer Res.*, **37**, 1450.

NEWLANDS, E.S., BLACKLEDGE, G., SLACK, J.A. and 4 others (1985). Phase I clinical trial of mitozolomide. *Cancer Treat. Rep.*, **69**, 801.

NIAS, A.H.W. (1985). Radiation and platinum drug interaction. *Int. J. Radiat. Biol.*, **48**, 297.

SINCLAIR, W.K. & MORTON, R.A. (1966). X-ray sensitivity during the cell generation cycle of cultured chinese hamster cells. *Radiat. Res.*, **29**, 450.

STEVENS, M.F.G., HICKMAN, J.A., STONE, R. and 4 others (1984). Antitumour imidazotetrazines. 1. Synthesis and chemistry of 8-carbamoyl-3-(2-chloroethyl)imidazo [5, 1-*d*]-1,2,3,5-tetrazine-4($^3$H)-one, a novel broad-spectrum antitumour agent. *J. Med. Chem.*, **27**, 196.

TERASIMA, T. & TOLMACH, L.T. (1963). Variations in several responses of HeLa cells to X-radiation during the division cycle. *Biophys. J.*, **3**, 11.

TOFILON, P.J., WILLIAMS, M.E. & DEEN, D.F. (1984). The effects of X-rays on BCNU-induced DNA crosslinking. *Radiat. Res.*, **99**, 165.

WALKER, M.D. & GEHAN, E.A. (1976). Clinical studies with malignant Gliomas and their treatment with nitrosoureas. *Cancer Treat. Rep.*, **60**, 713.

WHEELER, K.T., DEEN, D.F., WILSON, C.B., WILLIAMS, M.E. & SHEPPARD, S. (1977). BCNU-modification of the *in vitro* radiation response in 9L brain tumour cells of rats. *Int. J. Radiat. Oncol. Biol. Phys.*, **2**, 79.


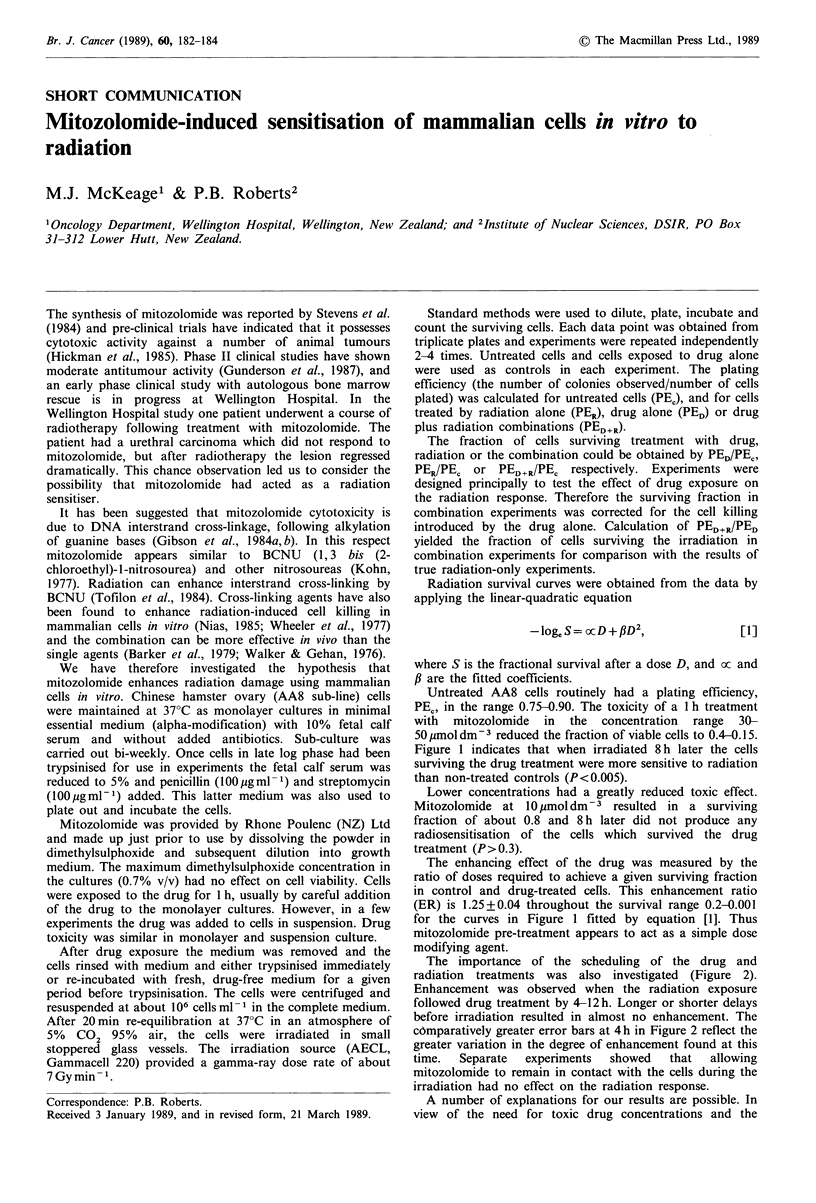

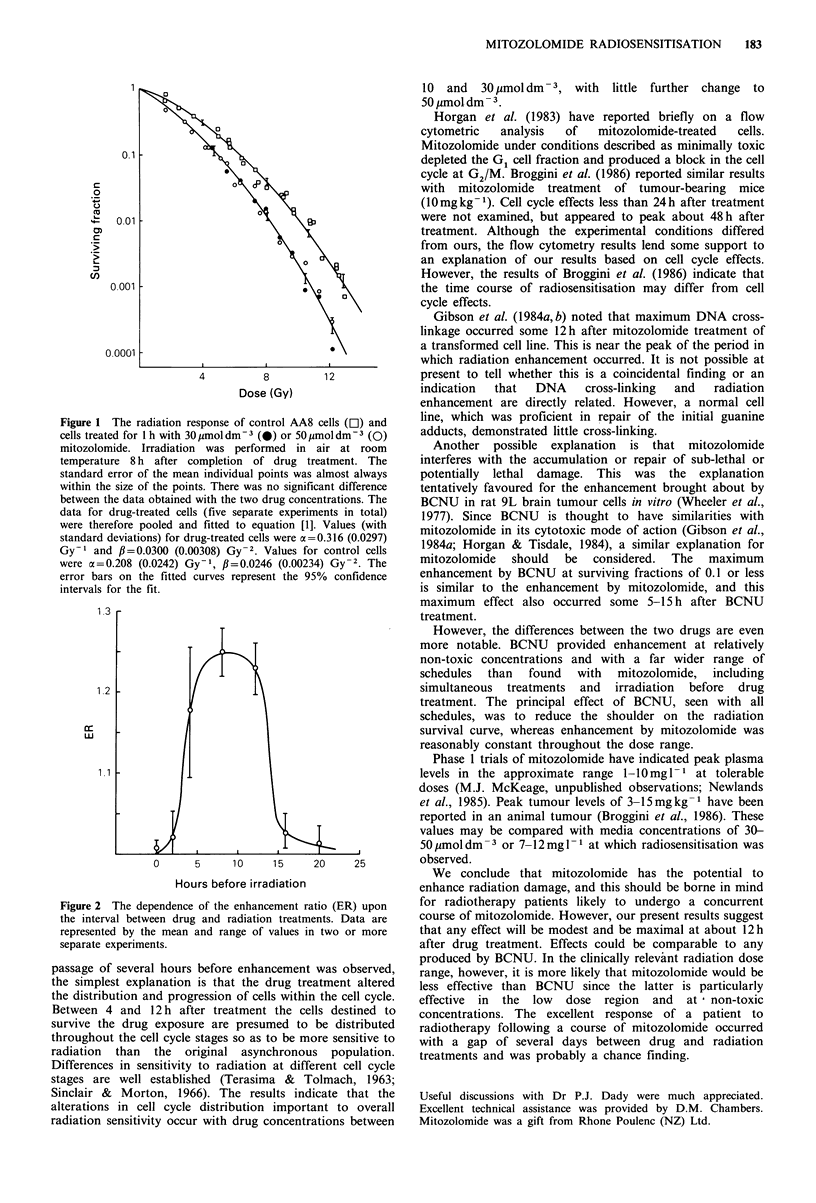

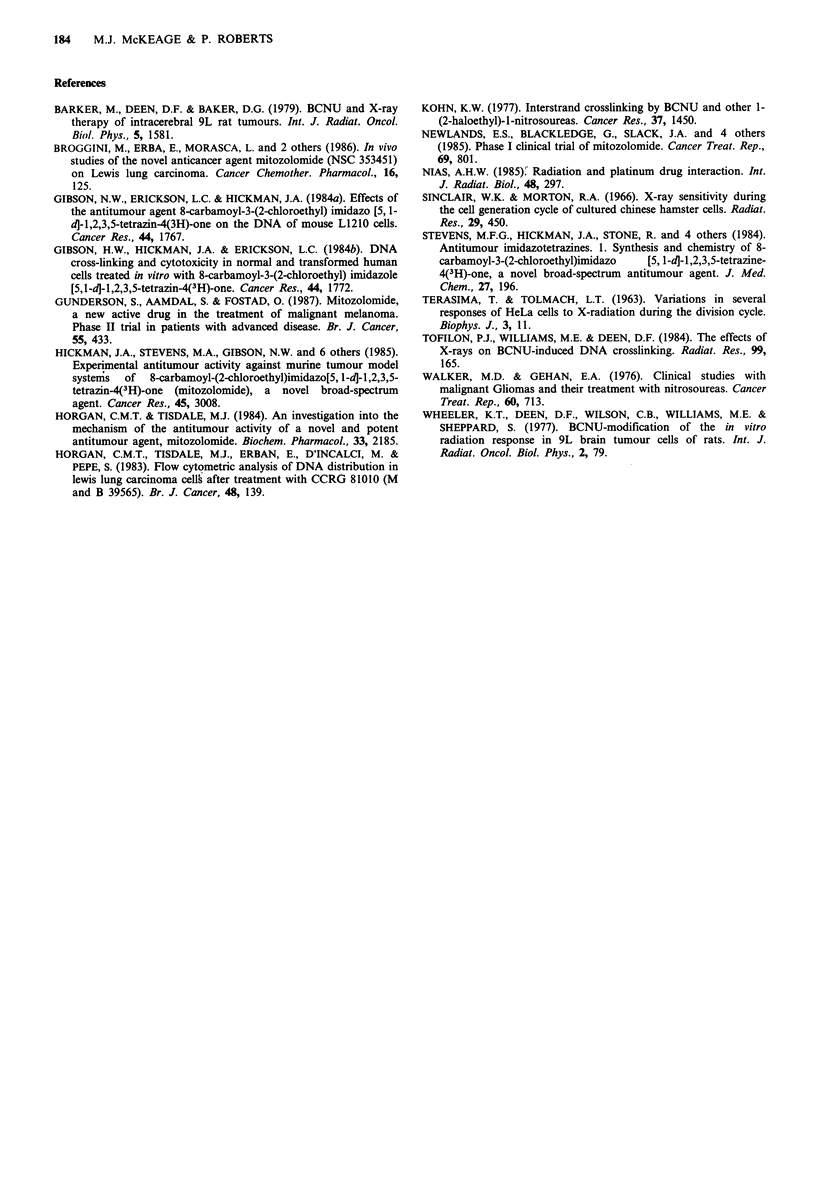


## References

[OCR_00311] Barker M., Deen D. F., Baker D. G. (1979). BCNU and X-ray therapy of intracerebral 9L rat tumors.. Int J Radiat Oncol Biol Phys.

[OCR_00316] Broggini M., Erba E., Morasca L., Horgan C., D'Incalci M. (1986). In vivo studies with the novel anticancer agent mitozolomide (NSC 353451) on Lewis lung carcinoma.. Cancer Chemother Pharmacol.

[OCR_00322] Gibson N. W., Erickson L. C., Hickman J. A. (1984). Effects of the antitumor agent 8-carbamoyl-3-(2-chloroethyl)imidazo[5,1-d]-1,2,3,5-tetrazin-4(3 H)-one on the DNA of mouse L1210 cells.. Cancer Res.

[OCR_00328] Gibson N. W., Hickman J. A., Erickson L. C. (1984). DNA cross-linking and cytotoxicity in normal and transformed human cells treated in vitro with 8-carbamoyl-3-(2-chloroethyl)imidazo[5,1-d] -1,2,3,5-tetrazin-4(3H)-one.. Cancer Res.

[OCR_00334] Gundersen S., Aamdal S., Fodstad O. (1987). Mitozolomide (NSC 353451), a new active drug in the treatment of malignant melanoma. Phase II trial in patients with advanced disease.. Br J Cancer.

[OCR_00340] Hickman J. A., Stevens M. F., Gibson N. W., Langdon S. P., Fizames C., Lavelle F., Atassi G., Lunt E., Tilson R. M. (1985). Experimental antitumor activity against murine tumor model systems of 8-carbamoyl-3-(2-chloroethyl)imidazo[5,1-d]-1,2,3,5-tetrazin-4(3 H)-one (mitozolomide), a novel broad-spectrum agent.. Cancer Res.

[OCR_00347] Horgan C. M., Tisdale M. J. (1984). Antitumour imidazotetrazines--IV. An investigation into the mechanism of antitumour activity of a novel and potent antitumour agent, mitozolomide (CCRG 81010, M & B 39565; NSC 353451).. Biochem Pharmacol.

[OCR_00357] Kohn K. W. (1977). Interstrand cross-linking of DNA by 1,3-bis(2-chloroethyl)-1-nitrosourea and other 1-(2-haloethyl)-1-nitrosoureas.. Cancer Res.

[OCR_00361] Newlands E. S., Blackledge G., Slack J. A., Goddard C., Brindley C. J., Holden L., Stevens M. F. (1985). Phase I clinical trial of mitozolomide.. Cancer Treat Rep.

[OCR_00366] Nias A. H. (1985). Radiation and platinum drug interaction.. Int J Radiat Biol Relat Stud Phys Chem Med.

[OCR_00370] Sinclair W. K., Morton R. A. (1966). X-ray sensitivity during the cell generation cycle of cultured Chinese hamster cells.. Radiat Res.

[OCR_00375] Stevens M. F., Hickman J. A., Stone R., Gibson N. W., Baig G. U., Lunt E., Newton C. G. (1984). Antitumor imidazotetrazines. 1. Synthesis and chemistry of 8-carbamoyl-3-(2-chloroethyl)imidazo[5,1-d]-1,2,3,5-tetrazin-4(3 H)-one , a novel broad-spectrum antitumor agent.. J Med Chem.

[OCR_00382] TERASIMA T., TOLMACH L. J. (1963). Variations in several responses of HeLa cells to x-irradiation during the division cycle.. Biophys J.

[OCR_00387] Tofilon P. J., Williams M. E., Deen D. F. (1984). The effects of X rays on BCNU-induced DNA crosslinking.. Radiat Res.

[OCR_00392] Walker M. D., Gehan E. A. (1976). Clinical studies in malignant gliomas and their treatment with the nitrosoureas.. Cancer Treat Rep.

[OCR_00397] Wheeler K. T., Deen D. F., Wilson C. B., Williams M. E., Sheppard S. (1977). BCNU-modification of the in vitro radiation response in 9L brain tumor cells of rats.. Int J Radiat Oncol Biol Phys.

